# Capi-score: a quantitative algorithm for identifying disease patterns in nailfold videocapillaroscopy

**DOI:** 10.1093/rheumatology/keae197

**Published:** 2024-03-26

**Authors:** Borja del Carmelo Gracia Tello, Luis Sáez Comet, Gema Lledó, Mayka Freire Dapena, Miguel Antonio Mesa, Miguel Martín-Cascón, Alfredo Guillén del Castillo, Elena Martínez Robles, Carmen Pilar Simeón-Aznar, Jose Antonio Todolí Parra, Diana Cristina Varela, Genessis Maldonado Vélez, Adela Marín Ballvé, Jimena Aramburu Llorente, Laura Pérez Abad, Eduardo Ramos Ibáñez

**Affiliations:** Internal Medicine Department, Hospital Clínico Universitario Lozano Blesa, Zaragoza, Spain; Aragon Health Research Institute, Zaragoza, Spain; Department of Internal Medicine, Hospital Universitario Miguel Servet, Zaragoza, Spain; Department of Autoimmune Diseases, Hospital Clinic de Barcelona, Barcelona, Spain; Thrombosis and Vasculitis Unit, Complejo Hospitalario de Vigo, Pontevedra, Spain; Rheumatology Section, Clinica El Rosario, Medellin, Colombia; Internal Medicine, Hospital General Universitario José M Morales Meseguer, Murcia, Spain; Autoimmune Unit, Hospital Vall d’Hebron, Barcelona, Spain; Department of Internal Medicine, Hospital General Universitario La Paz, Madrid, Spain; Autoimmune Unit, Hospital Vall d’Hebron, Barcelona, Spain; Internal Medicine, Hospital Universitario y Politécnico La Fe, Valencia, Spain; Rheumatology Department, Hospital General de Medellín Luz Castro de Gutiérrez, Medellin, Colombia; Rheumatology Department, Vanderbilt University Medical Center, Nashville, TN, United States; Internal Medicine Department, Hospital Clínico Universitario Lozano Blesa, Zaragoza, Spain; Aragon Health Research Institute, Zaragoza, Spain; Internal Medicine Department, Hospital Clínico Universitario Lozano Blesa, Zaragoza, Spain; Internal Medicine Department, Hospital Clínico Universitario Lozano Blesa, Zaragoza, Spain; Computer Engineer, University of Zaragoza, Zaragoza, Spain

**Keywords:** nailfold videocapillaroscopy, systemic sclerosis, Raynaud’s phenomenon, Fast Track algorithm, software-based algorithm, quantitative

## Abstract

**Objectives:**

EULAR supports the use of nailfold videocapillaroscopy (NVC) for identifying disease patterns (DPs) associated with SSc and RP. Recently, EULAR proposed an easy-to-manage procedure, a so-called Fast Track algorithm, for differentiating SSc patterns from non-SSc patterns in NVC specimens. However, subjectivity among capillaroscopists remains a limitation. Our aim was to perform a software-based analysis of NVC peculiarities in a cohort of samples from SSc and RP patients and, subsequently, build a Fast Track–inspired algorithm for identifying DPs without the constraint of interobserver variability.

**Methods:**

NVCs were examined by 9 capillaroscopists. Those NVCs whose DPs were consensually agreed upon (by ≥2 out of 3 interobservers) were subsequently analysed using in-house–developed software. The results for each variable were grouped according to the consensually agreed-upon DPs in order to identify useful hallmarks for categorizing them.

**Results:**

A total of 851 NVCs (21 957 images) whose DPs had been consensually agreed upon were software-analysed. Appropriate cut-offs set for capillary density and percentage of abnormal and giant capillaries, tortuosities and haemorrhages allowed DP categorization and the development of the CAPI-score algorithm. This consisted of four rules: Rule 1, SSc *vs* non-SSc, accuracy 0.88; Rules 2 and 3, SSc-early *vs* SSc-active *vs* SSc-late, accuracy 0.82; Rule 4, non-SSc normal *vs* non-SSc non-specific, accuracy 0.73. Accuracy improved when the analysis was limited to NVCs whose DPs had achieved full consensus between the interobservers.

**Conclusion:**

The CAPI-score algorithm may become a tool that is useful in assigning DPs by overcoming the limitations of subjectivity.

Rheumatology key messages

- Although capillaroscopy is widely used for various pathologies, its analysis largely remains subjective.
- The CAPI-score algorithm represents a novel integration of automated quantitative metrics in capillaroscopy with the consensus expertise of seasoned capillaroscopists, establishing a new benchmark for accuracy in the classification of NVC patterns.
- The CAPI-score is a robust algorithm that not only differentiates between SSc and non-SSc patterns in NVC, but also accurately identifies specific stages of SSc.

## Introduction

Nailfold videocapillaroscopy (NVC) is a non-invasive tool for assessing microvascular pathology. NVC is currently the method of choice for analysing capillary abnormalities in autoimmune rheumatic diseases such as SSc [1]. Furthermore, NVC has also been shown to be useful in other diseases with significant microvascular involvement, such as diabetes mellitus, hereditary amyloidogenic transthyretin amyloidosis or pulmonary arterial hypertension [2–4]. Visual examination of NVC images requires expertise to identify and describe correctly those abnormalities in capillary architecture. There is, therefore, a non-negligible researcher-related bias [5, 6]. Recently, when a group of 5 expert capillaroscopists examined independently 1164 images to identify abnormalities, consensus of ≥4 examiners was achieved in 52% of cases only [7]. This means that there may be a 40% likelihood of having inaccurate interpretations in approximately half of the images examined by highly experienced capillaroscopists. Furthermore, the procedure is time-consuming, lasting >15 min. Software-based automated systems have been developed to overcome these limitations, which are not only able to detect structural abnormalities but can identify whether these are associated with the presence of giant capillaries, tortuosities, abnormal shapes, haemorrhages, or changes in density of microvascularization [7–9].

A step beyond regarding NVC application to CTDs was taken when a NVC-based algorithm was proposed for identifying SSc patterns. The so-called Fast Track algorithm was proposed by a highly experienced member of EULAR, and its reliability was subsequently tested by other experts in two EULAR meetings [10]. The Fast Track algorithm contributed an easy-to-manage categorization between SSc and non-SSc patterns. Three simple rules had to be applied: first, a capillary density of ≥7 capillaries per mm and the absence of giant capillaries points to a non-SSc pattern; second, a capillary density ≤3 capillaries per mm in combination with abnormal shapes or giant capillaries allows the identification of a SSc pattern, which would point to SSc-late in the presence of abnormal shapes; the last rule stated that those images not meeting the previous rules should be considered as non-SSc. Recently, additional progress has been made. A deep-learning system for assessment of microvascular abnormalities in NVC images has been applied for identifying SSc patterns [11]. The procedure is based on the analysis of capillary architecture abnormalities, basically by following the criteria proposed by the aforementioned Fast Track algorithm.

We followed the algorithm-based approach to identify the presence or absence of SSc patterns, taking advantage of our in-house–developed software that allows us to analyse capillaries on an individual basis. We hypothesized that the latter would permit us to construct a Fast Track–inspired algorithm to apply to NVC capillaroscopies subjected to automated analysis, using quantitative criteria to assign/discard SSc patterns. By doing this, not only would examiner-related bias be avoided, but misinterpretation of algorithm rules would be minimized. We not only pursued mimicking the Fast Track algorithm, but also aspired to distinguish between the normal and non-specific patterns, and to go further with SSc samples and stratify them into early, active or late SSc patterns.

## Methods

### Joint working approach

We collected NVC images corresponding to patients with CTDs and distributed them among expert capillaroscopists to obtain consensual verdicts regarding SSc patterns. Subsequently, we submitted those capillaroscopies that achieved expert consensus to automated analysis with our in-house–developed software [12], in order to identify those variables that behaved differently according to one consensual pattern or another.

### Collection of capillaroscopies

NVC images taken in the course of routine explorations of patients with SSc or RP were collected in nine Spanish and Colombian hospitals. The Spanish participants belonged to the Spanish Autoimmune Systemic Diseases Group (GEAS) and the Multidisciplinary Spanish Society of Systemic Autoimmune Diseases (SEMAIS). The Latin America participants belonged to the Colombian Association of Rheumatology (ASOREUMA), with a proven experience of >500 capillaroscopies having been performed. The digital video capillaroscopes used were the Dino-Lite CapillaryScope 200 PRO (Dino-Lite Europe, Almere, The Netherlands), Smart G-Scope (Genie Tech, Seoul, Republic of Korea), Inspectis Capillaroscope (Inspectis, Kista, Sweden), or Optilia Digital Capillaroscope (Optilia Medical, Vällingby, Sweden). Capillary abnormalities could be correctly identified using any of these devices. A ×200 magnification (or the closest one to that) was always recommended.

The software and its database, as well as the project of building a software-based algorithm, were approved by the Clinical Research Ethics Committee of Aragón (Spain). All patients whose images were analysed by both the trained physicians and the software signed an informed consent form indicating that they agreed to participate. All the images were properly anonymized before their assignment to expert capillaroscopists.

### Consensus among capillaroscopists: gold standard

Nine expert raters in capillaroscopy analysed manually the NVC images in order to classify them within one of the following categories according to EULAR criteria [13]: normal; non-specific; SSc-early; SSc-active; SSc-late (Supplementary Methods, available at *Rheumatology* online).

Each patient’s capillaroscopy was randomly assigned to 3 of the 9 participant examiners. These were always blinded according to the patient’s diagnosis. Consensus among examiners would be considered the gold standard in the subsequent steps to find software-assessed measurable variables able to define capillary patterns. Consensus was considered to occur when ≥2 out of 3 experts agreed in their assignment of capillary pattern. Total consensus, i.e. 100% agreement among examiners, was also reported.

### Software-based analysis of NVC images

Those capillaroscopies whose NVC images allowed examiners to achieve consensus when classifying them into a capillary pattern among those cited above were selected to be analysed by our in-house–developed software. This software was designed to count and classify nailfold capillaries, and consists of a database to store information organized according to finger and nailfold sector, other image collections for research and dataset elaboration, a web application for managing data, and a desktop application for capturing images and uploading the information. The software, which was trained with images taken with different capillaroscopes and developed according to accepted criteria for identifying and classifying capillary abnormalities [13, 14], has been comprehensively described and validated elsewhere [7, 12].

Those capillaroscopies consisting of <8 images were discarded before software analysis, even though examiners had previously achieved consensus regarding pattern assignment. The software-assessed variables were capillary density and percentage of giant capillaries, haemorrhages, tortuosities and abnormal capillaries, the latter being defined as branching, bushy or coiled capillaries, often originating from a single normal-sized capillary [15]. The software analysis returned the results of each patient’s capillaroscopy as aggregated statistics of measured variables (average, s.d., sum, minimum/maximum, median, interquartile range, among others). Histogram graphs of each variable were grouped according to the disease pattern previously assigned by the gold standard.

### Development of a Fast Track–inspired algorithm able to identify capillary patterns

Those variables used by Smith *et al.* to develop the Fast Track algorithm were initially selected [10]. Other variables whose values were identified as being differently distributed among gold standard–assigned patterns were also considered. The chosen variables were categorized in order to find optimal cut-off points for discriminating between/among categories. Avoiding overfitting was a priority, and those conditions securing good precision and recall values for all patterns were selected, even though global accuracy could be slightly reduced. The idea was to generate a decision tree that, step by step, would allow us to finally assign one of the following categories to each capillaroscopy: Normal; Non-specific; SSc-early; SSc-active; SSc-late.

### Algorithm performance statistical analysis

The accuracy of each step created to stratify categories was separately analysed, and the accuracy of the algorithm was subsequently assessed. First, the reliability of each step of the algorithm was assessed by building confusion matrices and calculating precision (positive predictive value) and recall (sensitivity) according to gold standard verdicts (consensus of ≥2 experts) [16]. Then, the step-by-step and overall match between the gold standard and the algorithm prediction was also determined. The same calculations were also performed considering only those capillaroscopies whose verdict by the gold standard achieved full consensus. SPSS 22.0 and EPIDAT 3.0 (Xunta de Galicia, Spain) softwares were used for these purposes.

## Results

### Consensus among capillaroscopists in determining SSc patterns

**Figure 1.**
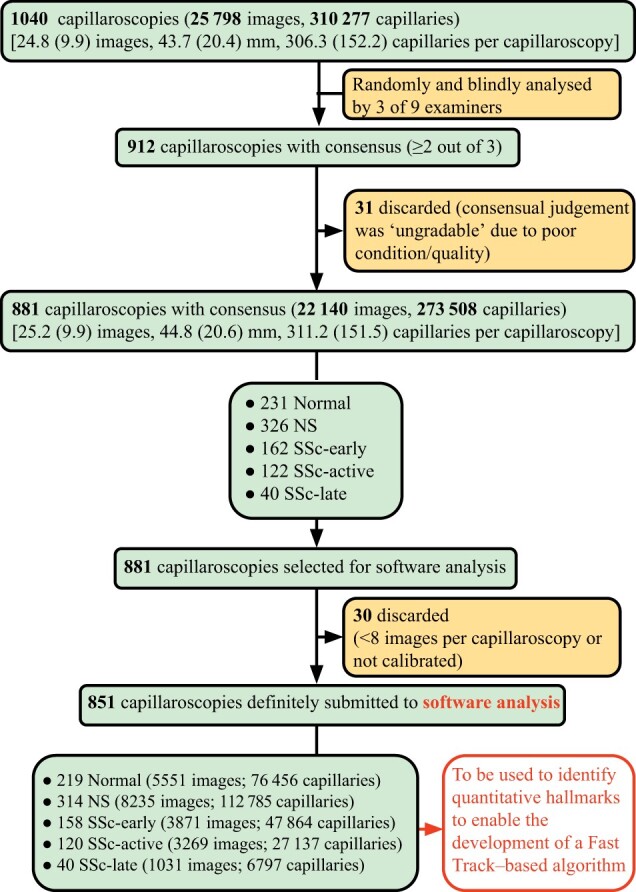
Flowchart diagram of the study. The sequence of steps in the study is shown, starting with the initially recruited NVCs and finishing with those finally selected to be software-analysed, which enabled us to develop a quantitative Fast Track–based algorithm. NS: non-specific; NVCs: nailfold videocapillaroscopies

A total of 1040 nailfold capillaroscopies were initially obtained from 1040 patients with SSc or RP (Fig. 1). A total of 25 798 images were analysed by the examiners [24.8 (9.9) images, 43.7 (20.4) mm, mean (s.d.) per capillaroscopy]. The total number of capillaries analysed was 310 277 [306.3 (152.2) per capillaroscopy]. Consensus was achieved in 912 out of 1040 (87.7%) capillaroscopies (Table 1). Both normal and SSc patterns, as well as the non-specific pattern, were well represented in the cohort, with >200 capillaroscopies being assigned to each one of these categories. Within SSc categories, only the late pattern had fewer than 100 consensual capillaroscopies. A total of 231 (26.22%), 326 (37%), 162 (18.39%), 122 (13.85%) and 40 (4.54%) capillaroscopies corresponding to normal, non-specific, SSC-early, SSC-active and SSc-late patterns, respectively, according to the gold standard, were submitted to software analysis. Total consensus (100% agreement among examiners) was observed in 328 (31.5%) capillaroscopies, 319 of whom could be assigned to a pattern (nine samples were unanimously considered unclassifiable).

**Table 1.** Consensus among capillaroscopists in identifying SSc patterns after visual examination of NVCs

Analysis	Consensus		Software accuracy	
	≥2 out of 3	3 out of 3	With consensus ≥2 out of 3	With consensus 3 out of 3
NVCs analysed, *n*	1040	1040	Non-SSc *vs* SSc	
NVCs with consensus, *n* (%)^a^	912^d^ (87.7)	328 (31.5)	0.88	0.89
Patterns with consensus reached, *n* (%)^b^				
Non-SSc	557 (61.0)	180 (54.9)		
Normal	231 (25.3)	86 (26.2)	SSc-early *vs* SSc-active *vs* SSc-late	
Non-specific	326 (35.7)	94 (28.7)	0.82	0.90
SSc	324 (35.6)	139 (42.4)		
Early	162 (17.8)	51 (15.6)	Normal *vs* non-specific	
Active	122 (13.4)	70 (21.3)	0.73	0.83
Late	40 (4.4)	18 (5.5)		
Ungradable^c^	31 (3.4)	9 (2.7)		

Consensus was considered to occur when ≥2 out of 3 or 3 out of 3 examiners agreed upon pattern assignment. The accuracy with which the software could identify patterns is shown on the right-hand side for comparison purposes.

a Percentage according to total number of analysed NVCs.

b Percentage according to total number of capillaroscopies with consensus reached.

c Due to poor image quality/condition. ^d^When excluding those consensual capillaroscopies considered ‘ungradable’, there were a total of 881 consensual capillaroscopies categorized as normal, non-specific or SSc patterns. NVCs: nailfold videocapillaroscopies.

### Definition of rules to categorize patterns on a dichotomous basis

Those software-assessed variables whose distribution among the capillary patterns consensually identified by the examiners was different enough to assist in pattern categorization were selected. These were: density, proportion of giant and abnormal capillaries, tortuosities, and haemorrhages. After 30 out of the 881 capillaroscopies whose pattern assignment had achieved consensus among examiners were discarded (because they consisted of fewer than eight images or lacked calibration), we finally obtained 851 software-analysed capillaroscopies (Fig. 1). When selected variables were grouped according to gold-standard consensual patterns, distinctive hallmarks could be identified. Appropriate cut-offs were then set to define rules able to stratify patterns on a dichotomous basis. Finally, quantitative metrics were computed to assess the quality of the rules.

#### First step: discrimination between SSc vs non-SSc patterns

Capillary density, percentage of giant capillaries and percentage of abnormal capillaries were useful in discriminating between non-SSc and SSc patterns.

#####  

An image is indicative of a SSc pattern whenever capillary density is ≤6 capillaries per mm and/or giant capillaries are detected and/or the proportion of abnormal capillaries is >10% (Supplementary Figs S1 and S2, available at *Rheumatology* online).

**Figure 2.**
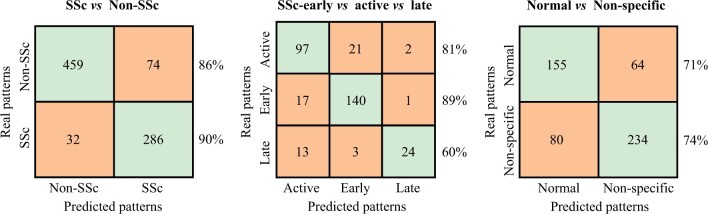
Confusion matrix showing matches and discrepancies between true and predicted disease patterns. All NVCs with consensus among capillaroscopists (gold standard: ≥2 out of 3 examiners, *n* = 851) were selected, and the proportion of matches (main diagonal of each matrix) and discrepancies (cells not on the main diagonal) when pattern assignment was performed by the gold standard (real patterns) or by software-based rules (predicted patterns) was annotated. Match percentage is indicated for each disease pattern. There were 30 capillaroscopies that were not analysed by the software, because they consisted of fewer than eight images or were not calibrated. NVCs, nailfold videocapillaroscopies

The accuracy corresponding to pattern recognition by applying this model was 0.88 (Table 2). The detailed description of matches between the gold standard and Rule 1 is shown in Fig. 2. Matches were always >85%. In this step, as well as in the forthcoming ones, performance metrics and proportion of matches improved when the analysis was restricted to those capillaroscopies whose pattern had been assigned with full consensus among examiners (Supplementary Table S1, Supplementary Fig. S3, available at *Rheumatology* online).

**Table 2.** Performance metric values of the models built to discriminate among disease patterns

Disease categories to discriminate between	Recall (sensitivity)	Precision (PPV)	Accuracy
Non-SSc *vs* SSc			0.88
Non-SSc	0.86	0.93	
SSc	0.90	0.79	
SSc-early *vs* SSc-active *vs* SSc-late			0.82
SSc-early	0.89	0.85	
SSc-active	0.81	0.76	
SSc-late	0.60	0.89	
Normal *vs* non-specific			0.73
Normal	0.71	0.66	
Non-specific	0.75	0.79	

The models were built by analysing those NVCs whose pattern assignment was coincident in the assessment performed by ≥2 out of 3 examiners (*n* = 851). Capillaroscopies were grouped according to pattern type, and all variables were analysed in order to select cut-offs useful for discriminating between categories. The rules finally chosen to achieve this purpose are described in the text. NPV: negative predictive value; NVCs: nailfold videocapillaroscopies; PPV: positive predictive value.

#### Second step: further discrimination within SSc patterns (early vs active vs late)

In order to achieve this goal, the same strategy followed in the first step was applied, now restricting the analysis to those capillaroscopies considered by consensus to respond to any SSc pattern. Supplementary Figs S4–S6 and Supplementary Fig. S2, available at *Rheumatology* online, show those variables allowing the setting of cut-offs helpful for distinguishing between the three SSc categories. The best results in terms of pattern recognition were obtained when two hierarchical rules inspired by these variables were followed (so-called by us Rule 2 and Rule 3).

#####  

When density is ≥5 capillaries per mm, the pattern is SSc-early, unless giant capillaries are ≥10% or abnormal capillaries are ≥5%. In such cases, the pattern is SSc-active (Supplementary Fig. S4, available at Rheumatology online).

#####  

When density is <5 capillaries per mm, the pattern is, initially, SSc-late. However, if either giant capillaries are ≥33% or abnormal capillaries are ≤7%, the pattern becomes SSc-active. Yet, regardless of these conditions, if ≤7% of the capillaries are giant or ≥15% of the capillaries are abnormal, the pattern is still SSc-late (Supplementary Figs S5 and S6, available at Rheumatology online).

The performance metrics and proportion of matches obtained by applying the aforementioned rules to consensus and full-consensus capillaroscopies are shown in Table 2 and Fig. 2, and Supplementary Table S1 and Supplementary Fig. S3, available at *Rheumatology* online, respectively. The accuracy was 0.82 and matches were >80% except for the late pattern. When the analysis was limited to those samples with full consensus, the accuracy increased to 0.90 and matches were >80% and >85% for the late pattern and the early and active patterns, respectively.

#### Third step: further discrimination within non-SSc patterns (normal vs non-specific)

Accomplishment of the last step was less straightforward, since the non-specific pattern is not well defined. After examining all variables and testing cut-offs, the best categorization was performed according to density and proportion of tortuosities, haemorrhages and abnormal capillaries (Supplementary Fig. S7, available at *Rheumatology* online). The following rule is proposed:

#####  

When density is >6 capillaries per mm, the pattern is non-specific when one or more of the following conditions are met: percentage of tortuosities is ≥20%; presence of haemorrhages; percentage of abnormal capillaries ≥2%. Otherwise, the pattern is normal (Supplementary Fig. S7, available at Rheumatology online).

Matches between true and predicted patterns were always >70%, and accuracy was 0.73 (Table 2, Fig. 2). Matches and performance metrics improved when restricting analysis to full-consensus capillaroscopies, with matches >75% and accuracy increasing to 0.83 (Supplementary Table S1, Supplementary Fig. S3, available at *Rheumatology* online).

### Development of the Fast Track–inspired CAPI-score algorithm

**Figure 3.**
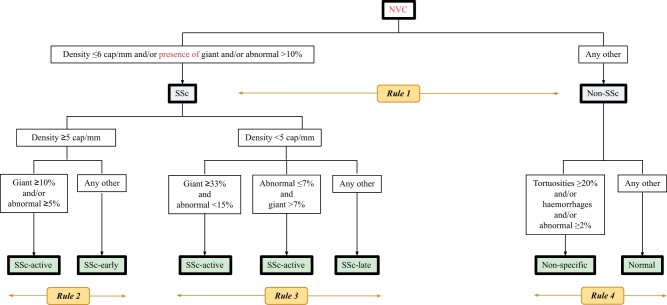
CapiScore algorithm for discriminating between SSc disease patterns. After grouping by disease pattern, all NVCs with consensus among capillaroscopists (≥2 out of 3 examiners, *n* = 881) were examined using the software. Thirty of them were discarded because they consisted of fewer than eight images or were not calibrated. Taking the remaining 851 capillaroscopies, cut-offs of variables that best discriminated between patterns were selected, and a series of rules for categorization were proposed: first, between SSc and Non-SSc patterns; second, within SSc, between SSc-early, SSc-active and SSc-late patterns; finally, within Non-SSc, between Normal and Non-specific patterns. cap, capillaries; NVCs, nailfold videocapillaroscopies

The four rules defined above were merged in order to construct our so-called CAPI-score algorithm, inspired by the aforementioned Fast Track algorithm (Fig. 3). By doing this, the software-based assessment of capillary density and proportion of giant capillaries, abnormal capillaries, haemorrhages and tortuosities, will allow us to assign a pattern to any NVC sample. Images representative of each one of the different disease patterns are shown in Supplementary Fig. S8, available at *Rheumatology* online.

## Discussion

NVC serves as an indispensable tool in the identification of microvascular changes, especially in diseases like SSc and RP. The value of NVC is not just restricted to its diagnostic utility but also extends to monitoring disease progression and therapeutic response [15]. Given the importance of NVC, reducing bias and enhancing the precision of interpretations is critical. The development of the Fast Track algorithm provided a comprehensive attempt in this direction [15]. We have tried to go one step beyond by minimizing subjectivity as much as possible. Subjectivity is not just a mere artifact of the process but often is the root cause of inter-observer variations. The problem is not limited to inexperienced examiners. A group of capillaroscopists was reported to achieve consensus not higher than 70% after having received comprehensive training [17].

Our approach was two-pronged. First, we relied on expert consensus, and second, we employed in-house–developed software to analyse the NVC images in an unbiased manner. This combination not only allowed for a more quantitative analysis but also minimized researcher-related bias and any potential misinterpretation of the algorithm rules. Furthermore, unlike other assessment methods, ours allows specialists to analyse capillaries individually. The mean number of capillaries-per-patient individually examined by the software, in the range of 300, provides additional reliability. The robustness of our approach was evident in the results. We could discern clear patterns and thresholds that could predict specific disease conditions with high precision. Our multi-step CAPI-score algorithm showed significant promise in discriminating between non-SSc *vs* SSc patterns and then further stratifying the SSc patterns.

One of the hallmarks of our software is that it is not only able to detect each capillary separately but also to identify its form and determine its size [12]. By doing so, the odds of obtaining dissenting verdicts when either expert capillaroscopists or the software assign patterns to NVC images are notably reduced. The results of the current study regarding performance metrics and proportion of matches between the gold standard and the software are encouraging and support this claim. Furthermore, the fact that the matching between both systems undoubtedly increased when the analysis was restricted to those NVC images that had achieved full consensus among examiners, further substantiates this notion. The rules that we proposed are along the same lines as those previously suggested by Smith *et al.* [12]. The mere presence of giant capillaries is a reliable marker of SSc, as previously anticipated by these authors.

We also addressed further categorization within patients with SSc. This topic is relevant to daily clinical practice. Early, active and late are the three NVC patterns of microvascular damage that are being used during routine clinical assessments, since they represent distinct phases of disease evolution [18]. Microangiopathy is an early event that is not often accompanied by full clinical symptoms, but which may progress to the active or late forms. Such a transition is usually accompanied by internal organ involvement [19], and it has been shown to occur in 47% of SSc patients after 5 years of follow-up [20]. Thus, SSc pattern assignment may condition therapeutic attitudes and follow-up routines. Our findings in this context were also in agreement with those of Smith *et al.*, in that low density was frequently associated with the late SSc pattern [15]. Nevertheless, we provide additional tools for further categorizing the status of patients with SSc. In our hands, low densities could also be associated with the SSc-active pattern, especially when the presence of giant capillaries is more evident than that of abnormal shapes. Densities above 5 capillaries per mm would strongly suggest the presence of early patterns, except in the presence of elevated proportions of giant capillaries and/or abnormal shapes, both occurrences being suggestive of active patterns. These structural hallmarks able to distinguish between SSc patterns are in agreement with those long since described [21]. Our rules regarding pattern recognition according to capillary density are in line with those found by Smith *et al.* in their Fast Track algorithm [10]. The slight differences observed in the capillary density cut-offs to discriminate between SSc and non-SSc patterns or to identify SSc-late patterns may be due to the inclusion of additional variables to assist in the categorization.

We also wanted to make progress in the distinction between normal and non-specific capillary patterns among those NVCs initially categorized within the non-SSc group, since patients presenting with the latter deserve a close follow-up to monitor future evolution to SSc forms. Although not predictive of defined conditions [14, 22], non-specific abnormalities have been reported in association with primary RP (PRP), other autoimmune diseases, SLE, SS and APS, and have been defined as a pre-SSc signature [23]. Identifying non-specific patterns is challenging. We found that, in those NVCs presenting with neither giant capillaries nor large amounts of capillaries with abnormal shapes, the presence of haemorrhages or a high proportion of tortuosities is useful for predicting non-specific rather than normal patterns.

Our findings may contribute to improving NVC analysis. The CAPI-score algorithm can also be applied to visual examinations of NVCs, although in such cases the concern of subjectivity would remain. In fact, we envisage the current contribution as being part of a process aimed to overcome the limitation of subjectivity definitively, to predict not only presence/absence of SSc (and the further characterization of the latter) but disease patterns as well. Deep-learning systems may be useful for this purpose, and the first attempts that have been performed with these tools have yielded encouraging results. A successful discrimination of patients with SSc, patients with PRP and healthy controls has been recently reported by using deep-learning networks to detect microvascular abnormalities in NVC images [11]. The study focused on vessel density, shape, and apical width, although capillaries were not analysed on an individual basis. Nevertheless, the incorporation of other software-assessed variables may allow us to identify other clinically relevant diseases associated with nailfold microvascular damage in the future [2–4].

Our study had limitations. The relatively low number of patients presenting with the SSc-late pattern according to the examiners’ criteria precluded reliable assessment of the accuracy of such predictions. Nevertheless, when the analysis was limited to those NVCs with full consensus among examiners, reassuring results were obtained regarding the predictive ability of the CAPI-score algorithm in identifying this pattern. On the other hand, as mentioned, non-specific patterns are not well defined by their nature. Nevertheless, by using quantitative metrics, we were able to categorize them with reasonable accuracy.

In summary, we have developed a software-based algorithm for identifying patterns of nailfold microvascular damage in NVC images, and have tested its accuracy in a cohort of SSc and RP patients whose capillary patterns had been previously determined by expert capillaroscopists. The application of quantitative metrics not only contributed to reducing inter-observer subjectivity but allowed us to further characterize SSc and non-SSc patterns.

## Supplementary Material

keae197_Supplementary_Data

## Data Availability

The data underlying this article will be shared on reasonable request to the corresponding author.
