# Supplementary material for: Capi-score: a quantitative algorithm for identifying disease patterns in nailfold videocapillaroscopy
Source: Rheumatology (Oxford). 2024 Mar 26;63(12):3315–21. doi: 10.1093/rheumatology/keae197 (PMC11637479; doi:10.1093/rheumatology/keae197)
Supplement: keae197_Supplementary_Data [file keae197_supplementary_data.zip › keae197_Supplementary_Data/rhe-23-2564-File008.pdf]

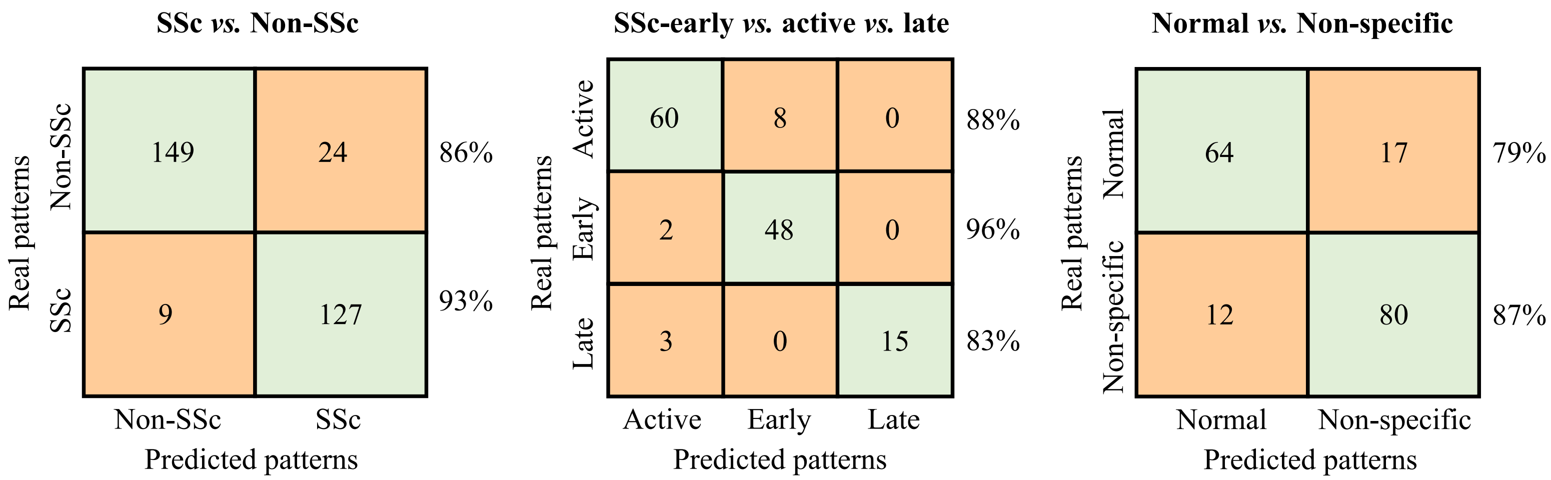
SSc vs. Non-SSc
Real patterns
Non-SSc
SSc
149
24
86%
9
127
93%
Non-SSc
SSc
Predicted patterns
SSc-early vs. active vs. late
Real patterns
Active
Early
Late
60
8
0
88%
2
48
0
96%
3
0
15
83%
Active
Early
Late
Predicted patterns
Normal vs. Non-specific
Real patterns
Normal
Non-specific
64
17
79%
12
80
87%
Normal
Non-specific
Predicted patterns
