# Supplementary material for: Capi-score: a quantitative algorithm for identifying disease patterns in nailfold videocapillaroscopy
Source: Rheumatology (Oxford). 2024 Mar 26;63(12):3315–21. doi: 10.1093/rheumatology/keae197 (PMC11637479; doi:10.1093/rheumatology/keae197)
Supplement: keae197_Supplementary_Data [file keae197_supplementary_data.zip › keae197_Supplementary_Data/rhe-23-2564-File014.docx]

**SUPPLEMENTAL METHODS**

**Criteria used by capillaroscopists to identify disease patterns**

(Smith et al, Autoimmun Rev 2020;19:102458)

● Normal pattern

Homogeneous distribution of hairpin-shaped capillaries as a ‘comb-like structure’, with a density of between 9 and 14 capillaries per mm (nevertheless, there is a wide intra- and inter-individual variability within a normal population, and tortuous and crossed capillaries may occur).

● SSc-early pattern

Few giant capillaries, few capillary micro-hemorrhages, no evident loss of capillaries and relatively well-preserved capillary distribution.

● SSc-active pattern

Frequent giant capillaries, frequent capillary micro-hemorrhages, moderate loss of capillaries, and absent or mild ramified capillaries.

● SSc-late pattern

Giant capillaries and micro-hemorrhages almost absent, severe loss of capillaries with extensive avascular areas, neo-angiogenesis with ramified/bushy capillaries and intense disorganisation of the normal capillary array.

● Non-specific patterns

These patterns do not fit with any of the above descriptions. A wide variety of presentations may be included in this category. Density is higher than that observed in scleroderma patterns.
