# Supplementary figures and images for: Capi-score: a quantitative algorithm for identifying disease patterns in nailfold videocapillaroscopy
Source: Rheumatology (Oxford). 2024 Mar 26;63(12):3315–21. doi: 10.1093/rheumatology/keae197 (PMC11637479; doi:10.1093/rheumatology/keae197)

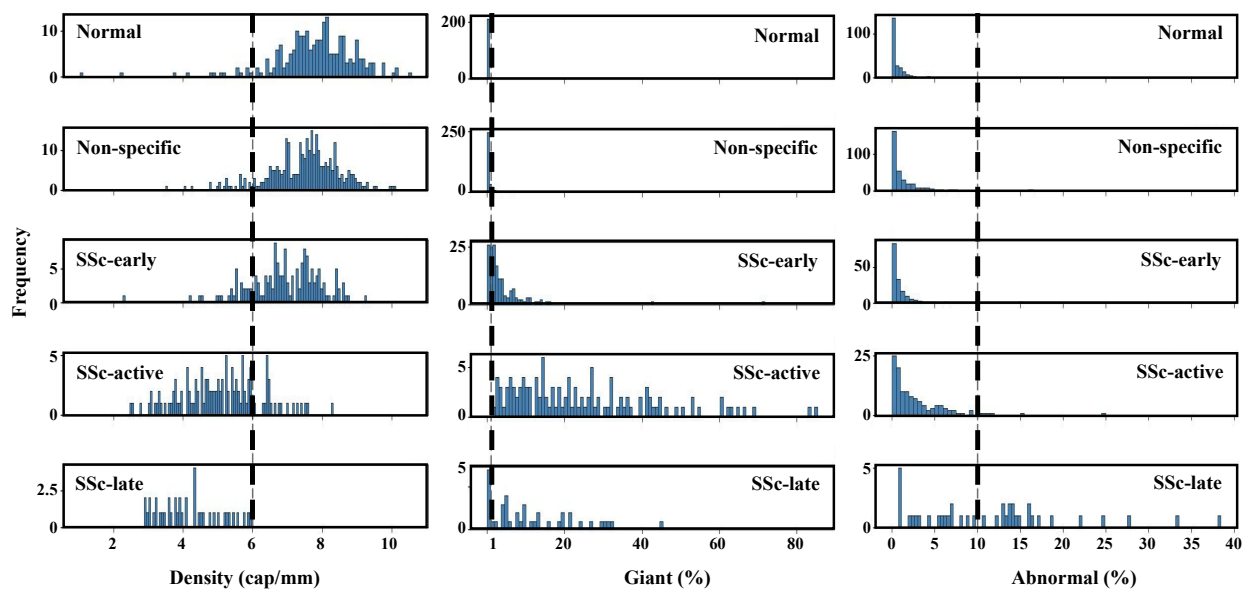

Supplement: keae197_Supplementary_Data [file keae197_supplementary_data.zip › keae197_Supplementary_Data/rhe-23-2564-File006.pdf]

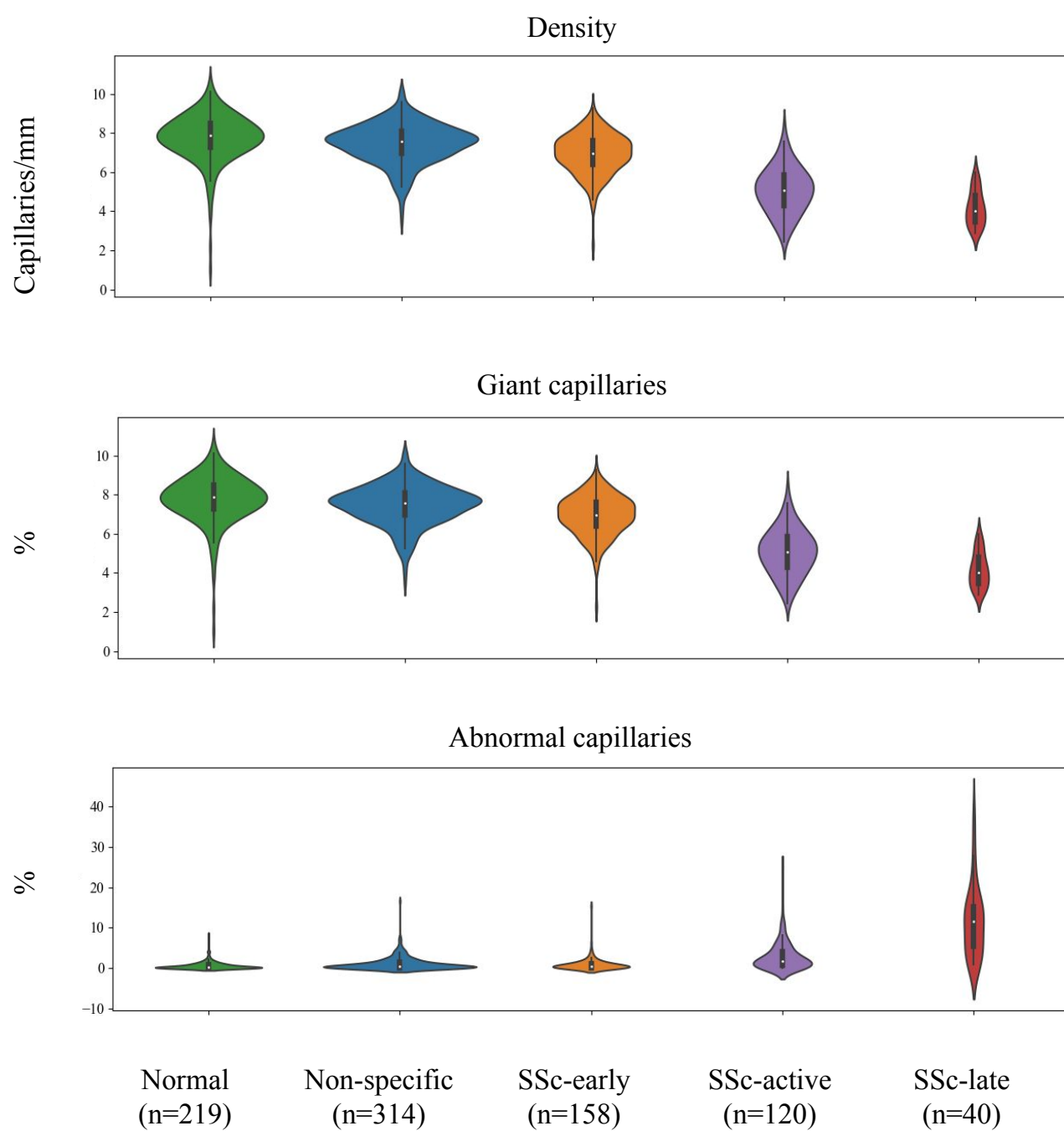

Supplement: keae197_Supplementary_Data [file keae197_supplementary_data.zip › keae197_Supplementary_Data/rhe-23-2564-File007.pdf]

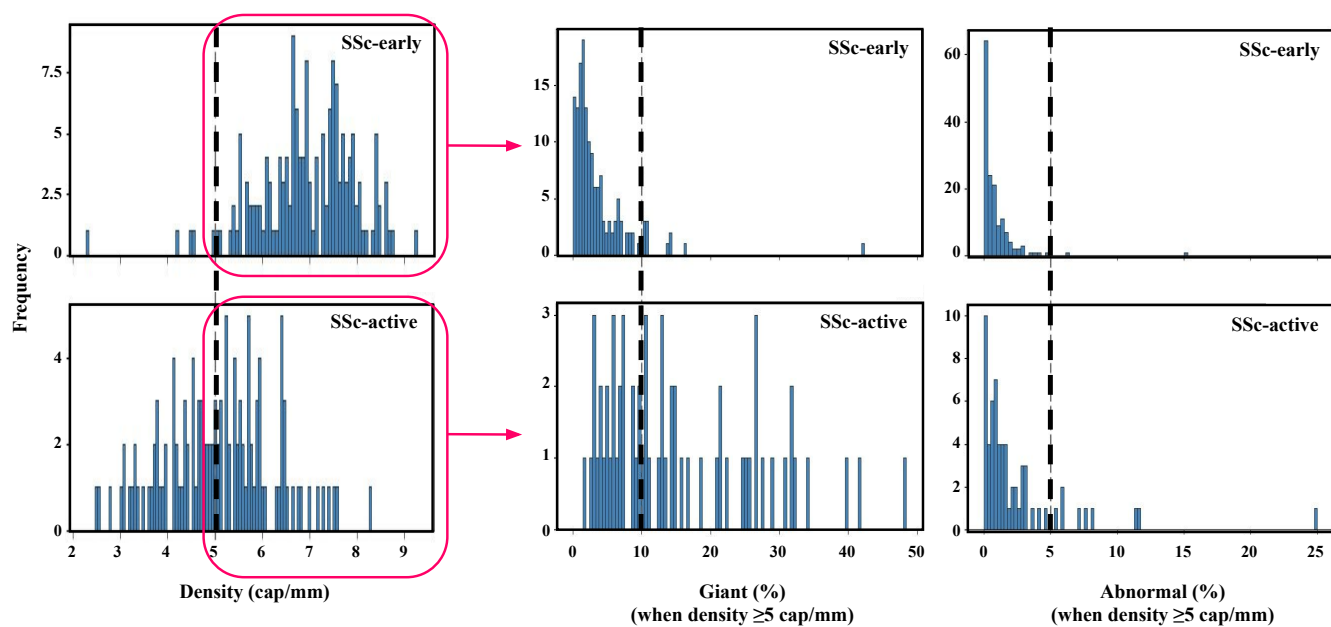

Supplement: keae197_Supplementary_Data [file keae197_supplementary_data.zip › keae197_Supplementary_Data/rhe-23-2564-File009.pdf]

**A**

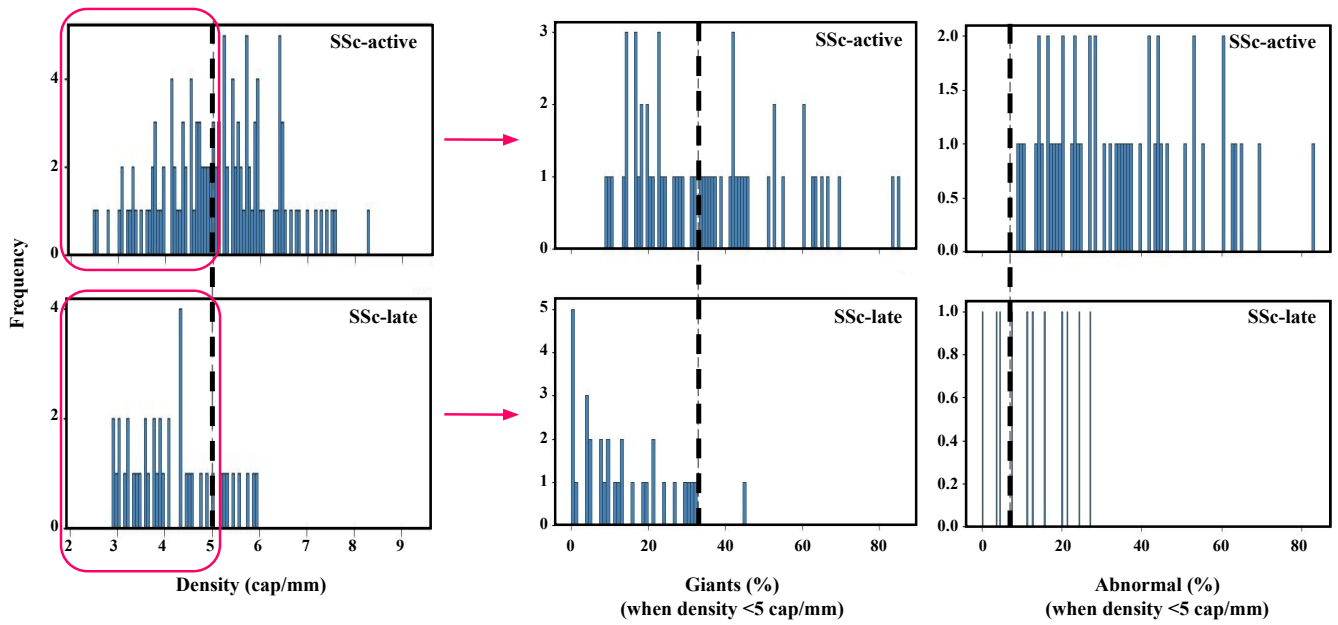

Supplement: keae197_Supplementary_Data [file keae197_supplementary_data.zip › keae197_Supplementary_Data/rhe-23-2564-File010.pdf]

## B

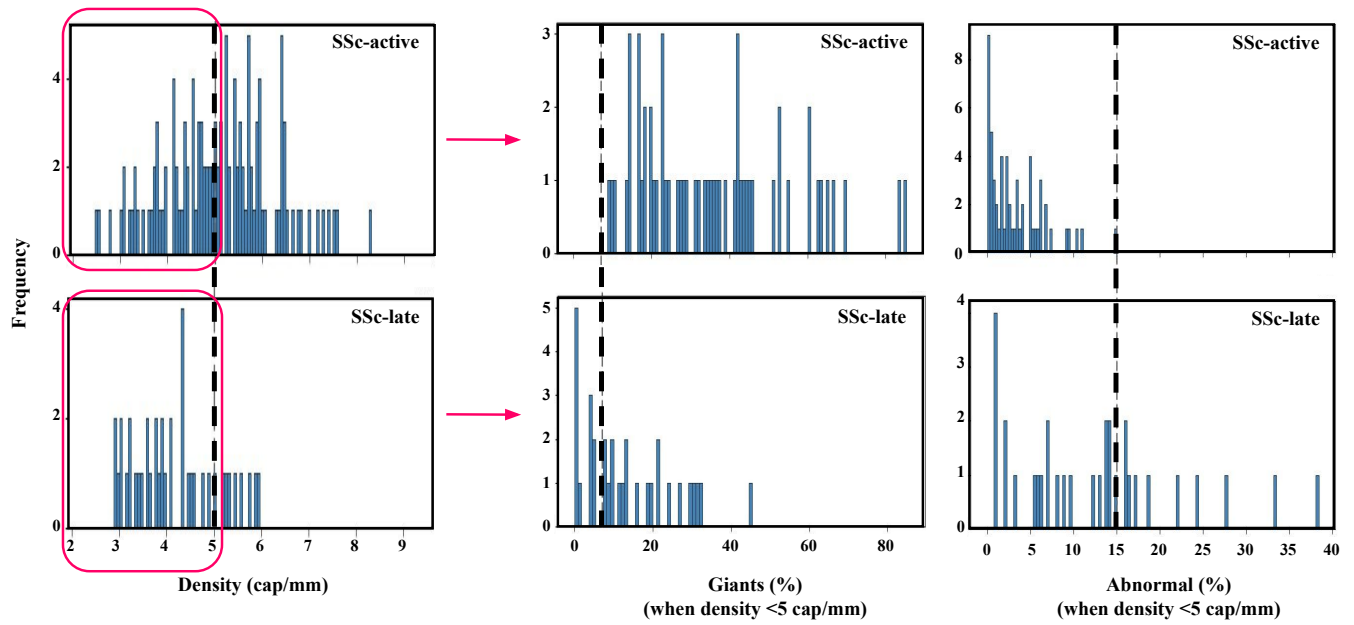

Supplement: keae197_Supplementary_Data [file keae197_supplementary_data.zip › keae197_Supplementary_Data/rhe-23-2564-File011.pdf]

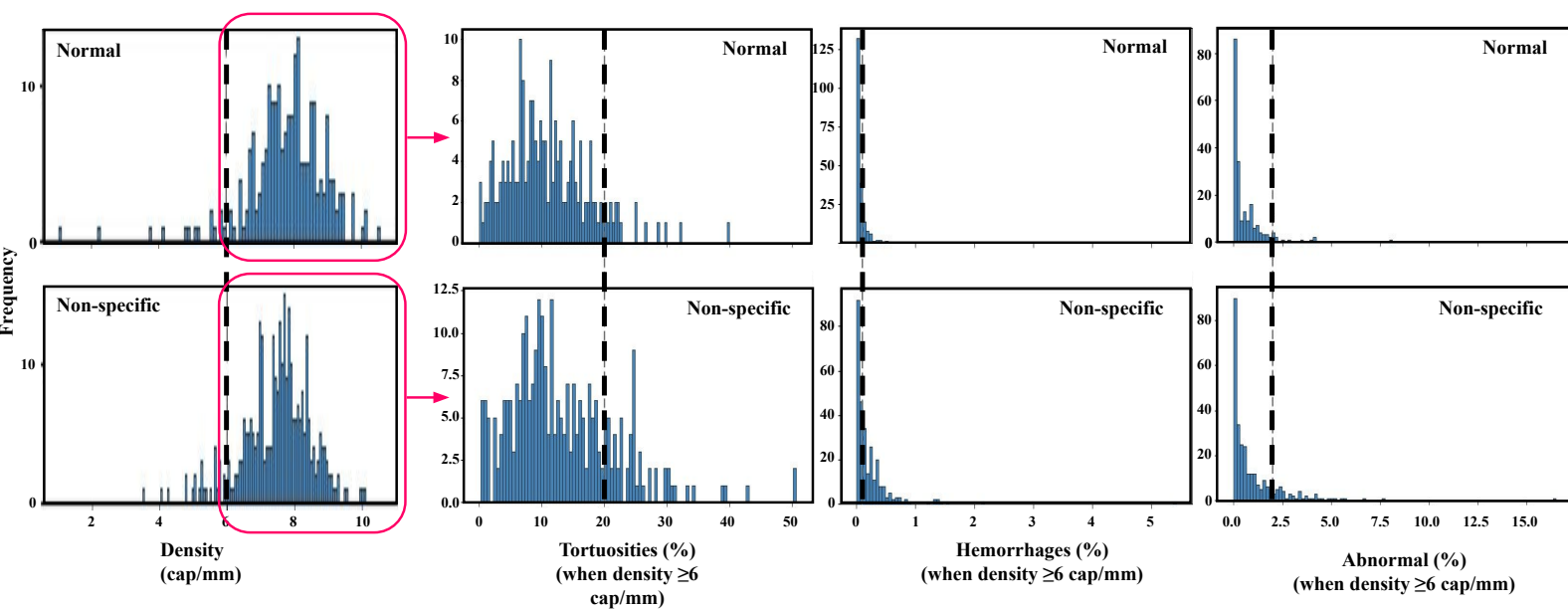

Supplement: keae197_Supplementary_Data [file keae197_supplementary_data.zip › keae197_Supplementary_Data/rhe-23-2564-File012.pdf]
